# Tin Oxide Encapsulated into Pyrolyzed Chitosan as a Negative Electrode for Lithium Ion Batteries

**DOI:** 10.3390/ma14051156

**Published:** 2021-03-01

**Authors:** Andrzej P. Nowak, Maria Gazda, Marcin Łapiński, Zuzanna Zarach, Konrad Trzciński, Mariusz Szkoda, Szymon Mania, Jinjin Li, Robert Tylingo

**Affiliations:** 1 Faculty of Chemistry, Gdańsk University of Technology, 80-233 Gdańsk, Poland; zuziaz696@gmail.com (Z.Z.); trzcinskikonrad@gmail.com (K.T.); mariusz.szkoda1@pg.edu.pl (M.S.); szymon.mania@pg.edu.pl (S.M.); 2 Faculty of Applied Physics and Mathematics, Gdańsk University of Technology, 80-233 Gdańsk, Poland; maria.gazda@pg.edu.pl (M.G.); marcin.lapinski@pg.edu.pl (M.Ł.); 3 Key Laboratory for Thin Film and Microfabrication of Ministry of Education, Department of Micro/Nano-electronics, Shanghai Jiao Tong University, Shanghai 200240, China; lijinjin@sjtu.edu.cn

**Keywords:** tin oxide, chitosan, lithium-ion battery

## Abstract

Tin oxide is one of the most promising electrode materials as a negative electrode for lithium-ion batteries due to its higher theoretical specific capacity than graphite. However, it suffers lack of stability due to volume changes and low electrical conductivity while cycling. To overcome these issues, a new composite consisting of SnO${}_{2}$ and carbonaceous matrix was fabricated. Naturally abundant and renewable chitosan was chosen as a carbon source. The electrode material exhibiting 467 mAh g${}^{-1}$ at the current density of 18 mA g${}^{-1}$ and a capacity fade of only 2% after 70 cycles is a potential candidate for graphite replacement. Such good electrochemical performance is due to strong interaction between amine groups from chitosan and surface hydroxyl groups of SnO${}_{2}$ at the preparation stage. However, the charge storage is mainly contributed by a diffusion-controlled process showing that the best results might be obtained for low current rates.

## 1. Introduction

In the last few decades, energy consumption has been growing year by year. It is mainly due to the significant increase in the use of mobile devices [1]. On one hand, it forces the world’s governments to look after renewable resources to be utilized as electrode materials for green and sustainable batteries. On the other hand, new electrode materials of enhanced electrochemical parameters, i.e., specific capacity, improved longevity and safety are required. Since 1991, anode electrodes in commercial rechargeable batteries have been mainly constructed from graphite [2]. However, graphite, with its theoretical capacity of 372 mAh g${}^{-1}$ no longer meets current requirements for mobile devices. Instead, negative electrode materials based on tin [3] or silicon [4] are taken into consideration. It is due to the fact that both of them exhibit higher theoretical capacity than graphite, 991 mAh g${}^{-1}$ for Li${}_{4.4}$Sn and 3579 mAh g${}^{-1}$ for Li${}_{15}$Si${}_{4}$ [5]. Unfortunately, the main issue with an electrode material based on tin or silicon is that both of them expand over 3 times during reaction with lithium ions. The fundamental reason behind the volume change is that the diffusion-induced stress is generated during lithiation and delithiation process [6]. The volume change lead to electrode’s structure damage, followed by loss of electric contact and capacity fading. To avoid these phenomena, different approaches were proposed, i.e., changing the size, morphology, incorporating with stress-accommodating phases to provide electronic conductivity [7]. Carbonaceous materials are the most common to be utilized as stress-accommodating phases, ensuring good mechanical stability, as well as good electrical conductivity. It is also important that the origin of the carbon source is vast and abundant. In recent years, many renewable organic materials have been investigated as potential sources of carbon in battery applications, i.e., starch [8,9], corn [10], rice husk [11], and cellulose [12]. The common feature of biomass-based electrode materials is that they belong to the hard carbon group exhibiting the higher reversible capacity and better cycling performance than graphite [13].

Chitosan is a polysaccharide composed of randomly distributed β(1→4)-linked d-glucosamine and *N*-acetyl-d-glucosamine units in a linear polymer structure. It is produced in a deacetylation process of chitin occurring in the exoskeleton of shellfish and insects, as well as in the cell walls of fungi [14]. Chitin and its derivatives, as well as the materials derived from them, are bioavailable, biocompatible, biodegradable, biofunctional, and environmentally friendly [15]. These features determine a high potential of these biopolymers in a range of applications in the biomedical, cosmetic, drug, environmental, and food industries [16]. Due to the lack of solubility of chitin in commonly used solvents, it is being converted into chitosan, which is soluble in dilute acid solutions, by means of deacetylation. Moreover, it is also possible to obtain chitosan solution devoided of acid residues by saturating the suspension of chitosan microcrystalline sediment with gaseous carbon dioxide ⁠[17], which significantly expands the application potential of chitosan. In addition, the presence of two functional groups in the molecule of chitosan, i.e., hydroxyl and amino, makes it possible to carry out many chemical and enzymatic modifications, which are frequently used in the design and construction of systems, e.g., for the immobilization and release of therapeutic compounds, as well as for obtaining water-soluble chitosan derivatives [18,19]. To the best of our knowledge, chitosan is known to be used as a binder for lithium-ion batteries in anode [20] or cathode [21] fabrication. It is due to fact that chitosan-based binders exhibit better electrochemical stability in comparison with ⁠commercially available polyvinylidene fluoride (pVDF). Chitosan was also used as a membrane’s component for proton batteries to increase ionic conductivity of such membrane [22]. However, there is a small number of research about utilization of chitosan as an active electrode material in battery applications. Li et al. synthesized nitrogen-doped carbon coated CoSnO${}_{3}$ via hydrothermal carbonization of chitosan anode material that was able to deliver a reversible capacity of 650 mAh g${}^{-1}$ after 50 cycles at 100 mA g${}^{-1}$ [23]. Prasanna et al. investigated milled silicon particles entraped into nitrogen-doped carbon of chitosan origin as an anode material in lithium-ion batteries [24]. Presented electrode material ehxibited discharge capacity of 942 mAh g${}^{-1}$ with coulombic efficiency of 97% after 50 cycles at 0.1 C rate. Recently, pyrolyzed chitosan was used as a source of hard carbons for sodium-ion batteries [25]. The authors showed that such anode material exhibited the average reversible capacity of 260 mAh g${}^{-1}$ at C/5 rate.

The main reason for the use of chitosan and tin oxide was the formation of hydrogen bonds between the amine group of chitosan and the hydroxyl groups on the surface of the tin oxide. Analogous strong interaction was observed by Lee et al. for the silicon anode with chitosan as a binder [26]. Thus, due to good adhesion between chitosan and SnO${}_{2}$, pyrolysis of such component could lead to the formation of yolk-shell structure preventing drastic volume changes during charging/discharging and improving electrochemical performance of negative electrode based on tin oxide/chitosan electrode material.

In this work, a new electrode material consisted of tin oxide embedded into carbon phase of chitosan origin has been investigated using scanning electron microscopy (SEM), X-ray diffraction (XRD) spectrometry, X-ray photoelectron spectroscopy (XPS), and electrochemical polarization methods.

## 2. Materials and Methods

Tin oxide particles were prepared by dissolution of metallic tin (Avantor Performance Materials Poland S.A., Gliwice, Poland) in 6 M HCl (Avantor Performance Materials Poland S.A., Gliwice, Poland) followed by adding chitosan (MMW, 75–85% deacetylated, Sigma Aldrich, St. Louis, MO, USA). Chitosan was obtained according to the procedure given in Reference ⁠[17]. Mixture was neutralized with 6 M NaOH (Avantor Performance Materials Poland S.A., Gliwice, Poland), followed by rinsing distilled water to remove any traces of chloride ions, and followed by heating in a horizontal tube-furnace (Czylok, Jastrzebie-Zdroj, Poland) under argon flow (Ar 5.0, 30 mL min${}^{-1}$). The pyrolysis time was equal to 2 h. In the further part of the text, the final product is designated as SnO_x_/CHI.

The X-ray powder diffraction (XRD) investigation on an Xpert PRO-MPD (Philips, Almelo, The Netherlands) diffractometer (Cu-Kα radiation (λ = 1.5404 Å) was used to analyze the phase composition of the pyrolyzed material in the range of 2θ = 20${}^{\circ }$–70${}^{\circ }$. Fityk software was used to process the diffraction pattern [27].

X-ray photoelectron spectroscopy (XPS) (Omicron NanoTechnology, Taunusstein, Germany) with an Mg-Kα X-Ray source (15 keV, 300 W) was used to determine the valence state and to analyze the environment of tin.

The electrode material, coated onto a Cu current collector (Schlenk Metallfolien GmbH&Co KG, Roth, Germany), consisted of SnO_x_/CHI (80 wt%), polyvinylidene fluoride (10 wt%) (PVdF, Solef 6020, Rheinberg, Germany), and the conductive additive (10 wt%) (Carbon Black Super P^®^, Timcal Ltd., Bodio, Switzerland).

The electrochemical experiments were carried out in two-electrode Swagelok® cells using multichannel battery testing system (ATLAS 0961 MBI, Gdansk, Poland). Lithium foil (99.9% purity, 0.75 mm thickness, AlfaAesar, Tewksbury, MA, USA) served both as a counter and a reference electrode. The electrolyte solution was 1 M LiPF${}_{6}$ in EC:DMC ratio 1:1 (LP30 Merck, Darmstadt, Germany), and glass fiber (Schleicher&Schüll, Dassel, Germany) was used as the separator. Cyclic voltammetry measurements (CV) and galvanostatic tests were performed on galvanostat/potentiostat (ATLAS 1361 MPG&T, Gdansk, Poland) within the potential range from 0.005 V to 3 V versus Li/Li${}^{+}$ with a scanning rate of 0.1 mV s${}^{-1}$ for CV. Galvanostatic tests were performed at different current densities in the sequence: 18 mA g${}^{-1}$ (5 cycles) → 36 mA g${}^{-1}$ (5 cycles) → 90 mA g${}^{-1}$ (5 cycles) → 180 mA g${}^{-1}$ (50 cycles), and, finally, 18 mA g${}^{-1}$ (5 cycles). The meaning “before” is for the electrode material that was not galvanostatically treated. The meaning “after” refers to the electrode material that was galvanostatically polarized.

## 3. Results and Discussion

### 3.1. XRD

**Figure 1:**
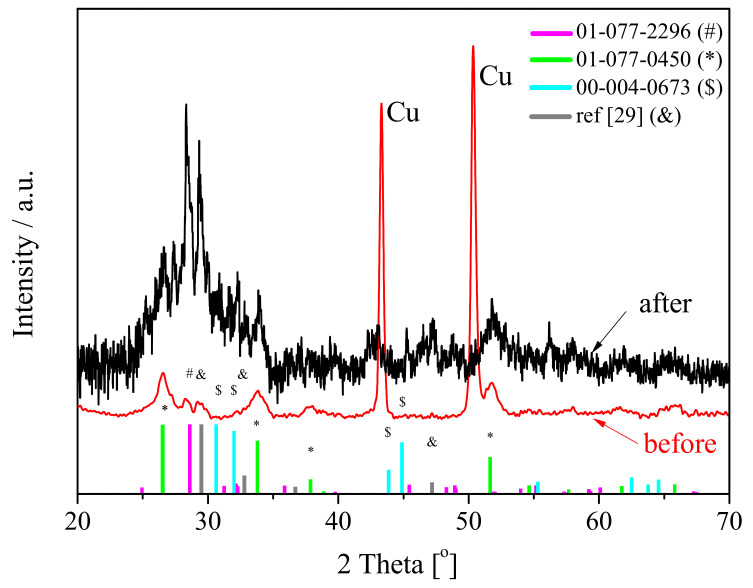
X-ray diffraction (XRD) of SnO_x_/CHI before (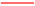) and after (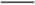) galvanostatic tests.

Figure 1 shows the XRD pattern of SnO_x_/CHI composite electrode material before (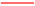) and after (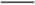) galvanostatic tests.

The XRD pattern for SnO_x_/CHI before cycles, apart from strong and narrow reflections originating from the support, consists of several relatively wide peaks. The strongest peaks seen at 2θ = 26.5${}^{\circ }$, 33.8${}^{\circ }$, and 51.7${}^{\circ }$ correspond to the (110), (101), and (211) planes of tin (IV) oxide. This oxide, which crystalizes in the tetragonal system [28] appears to be a majority phase. The reflections, which are seen at 28.4${}^{\circ }$ and 29.5${}^{\circ }$, may be attributed to the presence of orthorhombic SnO${}_{2}$ (01-077-2296) and tetragonal SnO [29], respectively. Therefore, XRD analysis evidences that tin is at +2 and +4 valency state in the system in a form of SnO and SnO${}_{2}$, respectively. The apparent size of tetragonal SnO${}_{2}$ crystallites estimated for diffraction reflection at 26.5°, from Scherrer equation [30], is 5 nm.

To estimate the weight fractions of SnO${}_{2}$ and SnO in the electrode material before galvanostatic tests, the Rietveld analysis of the XRD pattern was performed (see Figure S1). Due to the nano-crystalline nature of the oxides and related to that large widths of the XRD reflections, the quality of the refinement was relatively high (R_wp_ = 17%). Nevertheless, the amount of Sn(IV) was 76% wt. and Sn(II) was 24%. It seemed that, both on the surface and in the bulk of the material, there is more SnO${}_{2}$ than SnO. It is very likely due to surface oxidation of tin oxide.

After galvanostatic tests, one may see that the peaks’ position remained constant; however, the width of the reflections at 26.5${}^{\circ }$ and 33.8° diminished while reflections at 28.4${}^{\circ }$ and 29.5${}^{\circ }$ become more intense. It shows that after delithiation orthorhombic SnO${}_{2}$ and tetragonal SnO oxides are dominant. The estimated value of the apparent crystallite size of SnO${}_{2}$, from Scherrer equation, was 12 nm. It evidences that during the charging/discharging process SnO${}_{2}$ particles are forming aggregates (see Figure S2) as the value of the apparent crystallite size is increasing. Moreover, new reflections appeared in the 2 Theta range from 40${}^{\circ }$ to 50${}^{\circ }$. These signals, on the one hand, could be attributed to tin oxide, and, on the other hand, they might be originated from the solid electrolyte interphase (SEI) layer compounds that were formed during subsequent polarization cycles on the outer surface of the electrode material [31].

### 3.2. XPS

**Figure 2:**
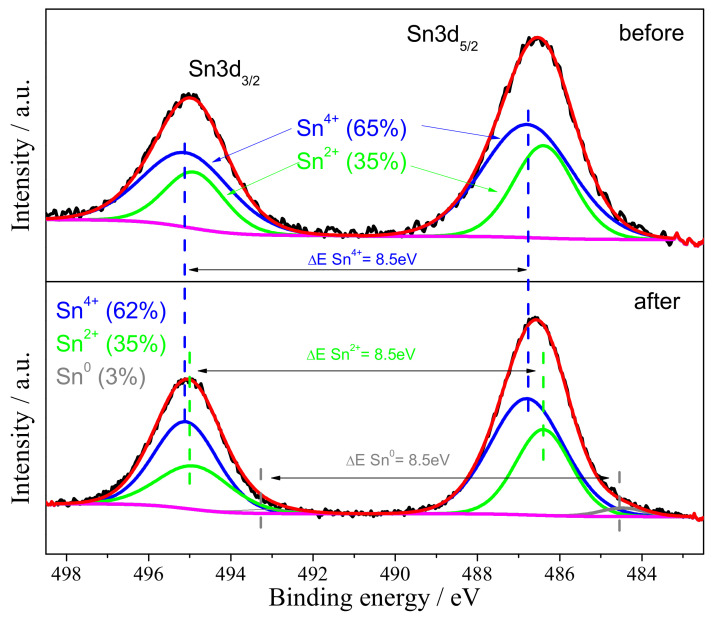
XPS spectra of Sn 3d core level of SnO_x_/CHI electrode material before and after galvanostatic charge/discharge.

To qualitatively identify the products of electrochemical reactions, XPS measurements were performed for the electrode material before and after galvanostatic charge/discharge. The XPS analysis was focused on the Sn 3d spectrum; see Figure 2.

On the basis of deconvolution of measured spectrum, it can be seen that the pristine electrode material consists of tin oxide at +4 and +2 valency state. The Sn 3d${}_{5/2}$ peaks maxima at 486.8 eV and 486.4 eV may be attributed to SnO${}_{2}$ and SnO, respectively. It is known that the binding energies of the Sn 3d${}_{5/2}$ peak of SnO and SnO${}_{2}$ are very similar [32]. The energy difference between Sn 3d${}_{5/2}$ peak and Sn 3d${}_{3/2}$ peak (ΔE) was 8.5 eV for both Sn${}^{4+}$ and Sn${}^{2+}$. The quantitative analysis of Sn content evidenced that amount of Sn(IV) was 65% wt., which was about 1.8 times higher than Sn(II). However, one should take into account that these results are only to be for comparison of tin at different oxidation levels. XPS analysis of the electrode material after the galvanostatic procedure showed the presence of an additional, low intense peak at 484.5 eV that was attributed to the metallic tin [33]. The amount of Sn${}^{0}$ was equal to 3%. It is very likely that not all tin was oxidized during the discharging process. On the other hand, no reflections of metallic tin were presented in the XRD patterns; however, it may be caused by the low sensitivity of XRD method. Moreover, the Sn 3d${}_{5/2}$ peak positions were almost the same as for the pristine material and were equal to 486.7 eV for Sn${}^{4+}$ and 486.3 eV for Sn${}^{2+}$. It evidenced that tin oxide underwent reversible process during charging/discharging. Besides, the weight ratio of Sn(IV) to Sn(II) was around 1.8 (62/35), which was almost the same as the one calculated for the pristine electrode material. The obtained results were consistent with the results obtained by XRD technique.

### 3.3. Electrochemistry

#### 3.3.1. Cyclic Voltammetry

**Figure 3:**
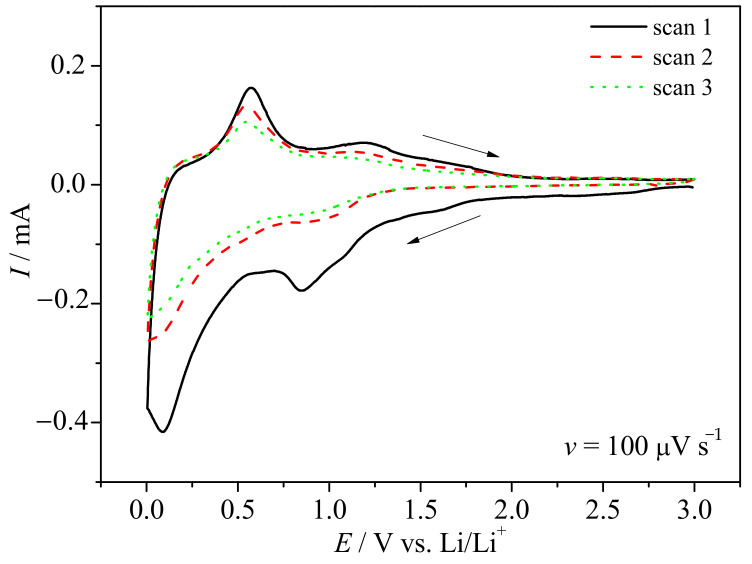
Cyclic voltammetry curves of SnO_x_/CHI electrode in LP30 at a sweep rate of *v* = 100 µV s${}^{-1}$ in the potential range 0.005–3.0 V versus Li/Li${}^{+}$.

Cyclic voltammetry curves of SnO_x_/CHI electrode material are shown in Figure 3.

**Figure 4:**
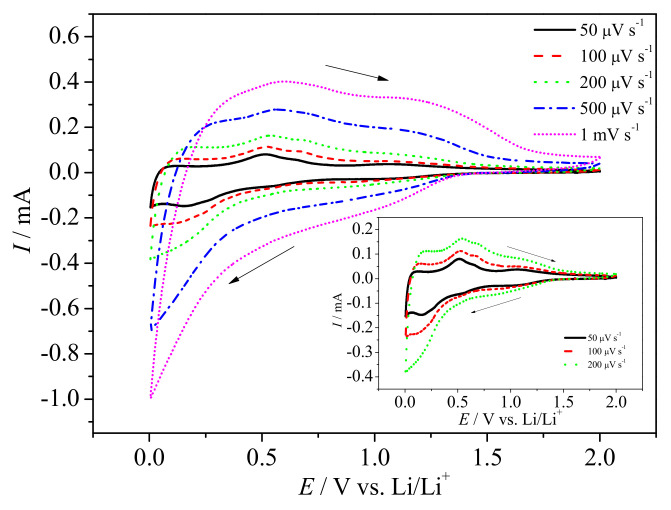
CV curves of SnO_x_/CHI electrode in LP30 at different sweep rates. Inset: Enlarged CV curves at sweep rates between 10 and 50 µV s${}^{-1}$. The potential range: 0.005–2.0 V versus Li/Li${}^{+}$.

Analyzing the curve obtained for the first cycle, one may see the cathodic current starts to increase at 1.7 V with a broad cathodic maximum at 0.86 V. It is very likely that two processes occur in that potential range. The first process is related with tin (IV) conversion reaction [34], while the second is attributed to the solid electrolyte interphase (SEI) formation [31]. One should take into account that the conversion reaction is not so simple. Recently, Fattakhova-Rohlfing et al. summarized that in the potential of conversion reaction the intermediate reaction occurs with the formation of Li_x_SnO${}_{2}$ [35]. In the studied case, further cathodic polarization led to another current growth starting at the potential E ≈ 0.53 V, following the second cathodic maximum at E = 0.09 V. This current growth is due to the presence of both carbonaceous matrix and tin oxide components in the electrode material. We assumed that two processes take place simultaneously: the first is lithiation to tin derived compound, and the second is lithium ions intercalation into the carbon phase. The final products of lithiation are Li${}_{8}$SnO${}_{6}$, Li${}_{2}$O, and Li_x_Sn of different lithium content [34]. During the oxidation process, three main anodic maxima are seen. The first, most intense, at ca. 0.6 V, may be attributed to initial delithiation of Li_x_Sn and deintercalation of Li_x_C. The second, at E ≈ 1.2 V, is related to further delithiation to metallic tin and Li_x_SnO${}_{2}$. The last, seen as a broad hump starting at E ≈ 1.55 V, is very likely associated with the oxidation of Sn${}^{0}$ followed by tin oxide (SnO_x_) formation. In the next cycles, a broad cathodic maximum between 0.8 V and 1.2 V is seen, followed by continuous cathodic current increase up to 0.005 V. The absence of cathodic maximum at 0.86 V evidences that this signal is attributed to SEI as it is believed to be formed in the first lithiation. Moreover, the lack of the cathodic maximum at 0.09 V may suggest that the alloy reaction is overlapped by lithium intercalation into the carbonaceous matrix. On the other hand, the obtained carbonaceous phase is amorphous and may not ensure proper diffusion path for lithium ions between carbon layers [36,37]. The cyclic voltammetry curves obtained at different scan rates are shown in Figure 4. One may see that the position and the shape of the current maxima depend on the sweep rate. It indicates that the charge storage mechanism is not only a diffusion-controlled process. To evaluate whether the electrode process is managed by the diffusion or surface mechanism, in its nature, the examination of scan rate dependence was performed. The total current response can be written in a form shown in Reference [38]:

(1) $$I\left(V\right)=k_{1}\cdot v+k_{2}v^{1/2},$$

where *I(V)* is the total current response at a given potential V, *k${}_{1}$·v* is attributed to capacitive behavior, and *k${}_{2}$·v${}^{1/2}$* is associated with the processes controlled by the diffusion. The constants *k${}_{1}$* and *k${}_{2}$* can be calculated from the plot *I(V)/v${}^{1/2}$ = f(v${}^{1/2}$)*. Knowing the values of these two parameters, one is able to quantify, at a given potential, the contribution of each mechanism in the total current response.

**Figure 5:**
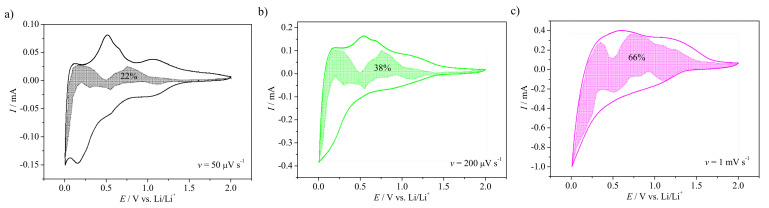
The fraction of the current response at (**a**) 50 µV s${}^{-1}$, (**b**) 200 µV s${}^{-1}$, and (**c**) 1 mV s${}^{-1}$ sweep rates regarding to the charge storage contribution. The shaded regions are associated with the capacitive currents.

The analysis of the charge storage contribution at sweep rates 50 µV s${}^{-1}$, 200 µV s${}^{-1}$, and 1 mV s${}^{-1}$, according to Equation (1), is given in Figure 5.

**Figure 6:**
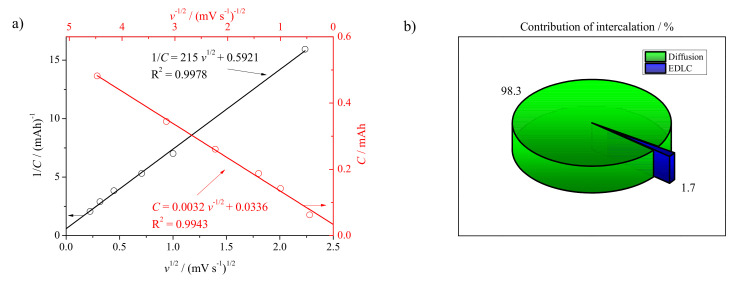
Plots of (**a**) (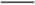) reciprocal specific capacitance versus square root of a sweep rate, (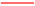) specific capacitance versus sweep rate, and (**b**) percentage of the intercalation process contribution.

The shaded regions in Figure 5a–c are attributed to the capacitance, and the area of those regions enlarges with the increase of the sweep rate. This increase of the capacitive contribution evidenced that, for low sweep rates, the charge transport is dominated by the intercalation process of lithium ions into the bulk material. Thus, diffusion of lithium ions seems to be a rate limiting step that affects the charge storage capability of the electrode material at higher current densities. It is very likely that, for higher current densities, the capacitance contribution related to the adsorption mechanism might be not sufficient to store as much charge as it could be achieved for the faradaic reaction. The second analysis method to distinguish between the intercalation process and the capacitive behavior was proposed by Trasatti [39]. The diffusion process is expected to show a linear relationship for *C = f(v${}^{-1/2}$)*, while the capacitance is linearly related to *v${}^{1/2}$*:

(2) $$1/C=a_{1}\cdot v^{1/2}+1/C_{TOT},$$

(3) $$C=a_{2}\cdot v^{-1/2}+C_{EDCL},$$

(4) $$C_{TOT}=C_{EDCL}+C_{D},$$

where *C*—the capacity calculated from CV curve (mAh), *v*—sweep rate (V s${}^{-1}$), *C${}_{TOT}$*—the total capacity [mAh g${}^{-1}$], *C${}_{EDCL}$*—the capacity originating from electrochemical double layer (mAh g${}^{-1}$), and *C${}_{D}$*—the specific capacity attributed to the diffusion-controlled process. The results obtained with Trasatti method indicate that SnO_x_/CHI electrode material stores charge by lithium-ion insertion mechanism; see Figure 6.

**Figure 7:**
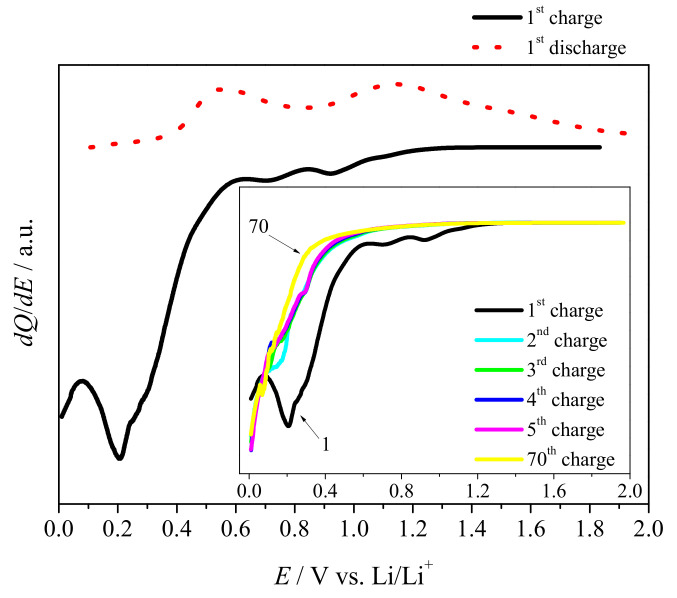
Differential capacity plot (dQ/dE) of lithiation and delithiation for the first cycle of SnO_x_/CHI electrode material. Inset: dQ/dE for cycles 1–5 and 70.

However, one should take into account that this method is valid if a linear relationship of *1/C = f(v${}^{1/2}$)* is fulfilled in the whole range of the sweep rate. In most cases it does not follow the trend [40]. Thus, the given approach showed that, for the analyzed range, the electrode material exhibited more diffusion dependence than than the capacitive one. The differential capacity plot for SnO_x_/CHI electrode material is shown in Figure 7.

During the first lithiation, one may clearly see three cathodic maxima at 0.9 V, 0.7 V, and 0.2 V attributed to the SEI formation, the SnO${}_{2}$ conversion reaction, and the lithium intercalation into a carbon phase, respectively. There is also a hump at ≈0.3 V that is ascribed to the alloy formation. The presence of the carbonaceous matrix seems to hinder redox activity originating from the inorganic part of the electrode material, especially for reduction process. On the oxidation curve, there are two anodic maxima at 0.55 V and 1.1 V, and a small hump at ≈0.6 V. The maximum at 0.55 V corresponds to the lithium deintercalation from the carbonaceous matrix, while the current peak at 1.1 V originates from the Li_x_SnO_y_ oxidation to Sn and SnO_x_ [35]. The hump at ≈0.6 V evidences Li_x_Sn delithiation process with the formation of Li_x_SnO_y_.

In the subsequence cycles (see Inset in Figure 7), cathodic maxima at 0.9 V and 0.7 V were not detected, which was confirmed by the results obtained from CV results showing that the SEI was formed in the first charging process. The disappearance of the current maximum at 0.7 V suggests that the conversion of tin oxide is an irreversible process. One may also see that, with the increasing number of cycles, the shape of lithiation curves changes, and no current maxima are detected. It is very likely due to fact that carbon phase is amorphous and affects lithium diffusion process into carbon layers, as well as lithium diffusion path to the tin compound.

#### 3.3.2. Galvanostatic Tests

**Figure 8:**
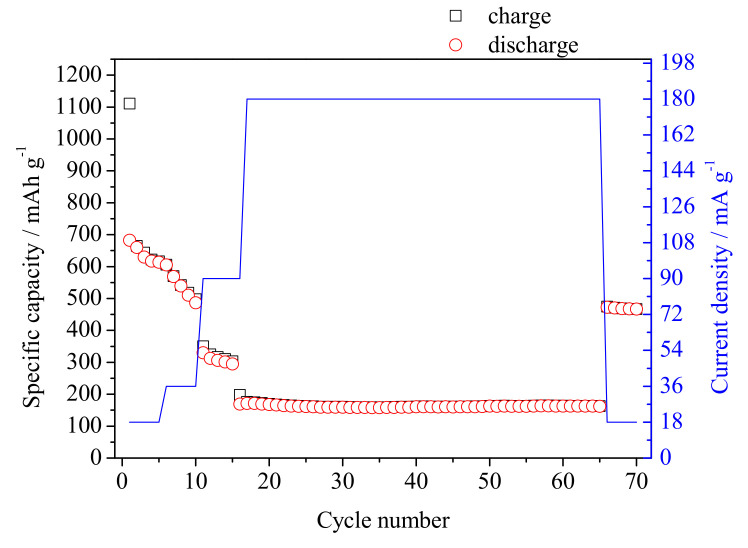
Specific capacity versus the cycle number of SnO_x_/CHI electrode material at different current rates.

The results of specific capacity calculated from galvanostatic charge/discharge process at different current densities are presented in Figure 8.

The specific capacity of the first lithiation and delithation was 1110 mAh g${}^{-1}$ and 682 mAh g${}^{-1}$, respectively. Such big difference is due to the two processes taking place during the first charging: the SEI formation and the conversion reaction. The capacity loss of 428 mAh g${}^{-1}$ is even higher than the theoretical specific capacity of graphite (372 mAh g${}^{-1}$) and shows that the material should be optimized before its utilization in practical applications. The specific capacity value of the fifth discharge of 613 mAh g${}^{-1}$ is 69 mAh g${}^{-1}$ lower in comparison with the value at the first discharge, which gives the capacity fading of 10% and is not acceptable performance of the electrode material for batteries. For the higher current rate (180 mA g${}^{-1}$), the specific capacity of the first and the last discharge was 171 mAh g${}^{-1}$ and 162 mAh g${}^{-1}$, respectively. The capacity retention for those 50 cycles was 95%. After changing the current density to 18 mA g${}^{-1}$, the specific capacity of the last cycle was 467 mAh g${}^{-1}$ with the capacity retention of the last five cycles of 98%. One may see that, with the increase of the number of cycles, the electrode material shows improved electrochemical behavior. However, it is shown that the specific capacity is affected by the applied current density and the higher density, and the lowest specific capacity value is obtained. It is consistent with the discussion raised about the results presented in Figure 4. It can be concluded that, for the SnO_x_/CHI electrode material, the diffusion-controlled processes have a major contribution in the overall specific capacity value and, thus, the charge storage process. It was evidenced that too high sweep rate value and too high current densities led to the decrease in the charge storage capacity values. The obtained SnO_x_/CHI composite stores charge mainly by the faradaic reaction between lithium ions and tin oxide. However, one should take into account that the pyrolysis temperature might have been too low to ensure high electronic conductivity at higher current density.

## 4. Conclusions

The electrode material consisting of pyrolyzed chitosan and tin oxide was fabricated to act as a negative electrode for lithium-ion batteries. Chitosan was used as a source of carbon as it is known to form hydrogen bonding with surface groups of tin oxide. It affected interactions between tin oxide and chitosane before pyrolysis and might have been a crucial factor on the electrochemical performances of the pyro ed composite electrode material. A solid-state physics technique analysis evidenced that SnO_x_/CHI consisted of a tetragonal crystal system of SnO${}_{2}$ and SnO that underwent structural changes after the charging/discharging with the formation of orthorhombic SnO${}_{2}$, while the structure of SnO became unchanged. The change of the crystallographic structure were coupled with the increase of the apparent crystallite size from 5 nm to 12 nm. XPS analysis evidenced the presence of SnO${}_{2}$ and SnO of similar Sn(IV) to Sn(II) ratio for electrode material before and after the charging/discharging process. Electrochemical tests showed that both SnO_x_ and the carbonaceous phase from pyrolyzed chitosan were active for lithium ion insertion. The redox couple activity was attributed to the conversion reaction of tin oxide and intercalation of lithium cations into a carbon matrix. The total charge storage with the utilization of SnO_x_/CHI electrode material was mainly originating from the diffusion-controlled processes. It was independently confirmed by the sweep rate dependences of voltammetric curves, as well as by the results obtained for low and high current densities during the galvanostatic mode. The specific capacity was equal to 467 mAh g${}^{-1}$ and 162 mAh g${}^{-1}$ at low (18 mA g${}^{-1}$) and high (180 mA g${}^{-1}$) current densities, respectively. The capacity retention of 98% for the last cycle suggests that the material is suitable for the energy storage and conversion systems where high currents are not required.

## Data Availability

The data presented in this study are available on request from the corresponding author.

## Supplementary Materials

The following are available online at https://www.mdpi.com/1996-1944/14/5/1156/s1, Figure S1: The Rietveld analysis of the xrd pattern obtained on the material not supported on the copper support, using SnO SG P4nmm (no. 129) and SnO${}_{2}$ SG P42mnm (no. 136) as starting data for the model. Figure S2: SEM image of SnO_x_/CHI surface before (a–c) and after (d–f) electrochemical measurements. Figure S3: CV curves of CHI, SnO${}_{2}$, and SnO_x_/CHI electrode in LP30 at 100 V s${}^{-1}$. The potential range: 0.005–2.0 V versus Li/Li${}^{+}$. Figure S4: CV SnO_x_/CHI electrode material at a different sweep rates. Current is reexpressed as a capacitance.
