# Supplementary material for: Tin Oxide Encapsulated into Pyrolyzed Chitosan as a Negative Electrode for Lithium Ion Batteries
Source: Materials (Basel). 2021 Mar 1;14(5):1156. doi: 10.3390/ma14051156 (PMC7957769; doi:10.3390/ma14051156)
Supplement: Supplementary file 1 [file materials-14-01156-s001.pdf]

Supplementary Materials: Tin oxide encapsulated into pyrolysed chitosan as a negative electrode for lithium ion batteries

Andrzej P. Nowak [1]*, Maria Gazda [2], Marcin Łapiński [2], Zuzanna Zarach [1], Konrad Trzciński [1], Mariusz Szkoda [1], Szymon Mania [1], Jinjin Li [3] and Robert Tylingo [1]

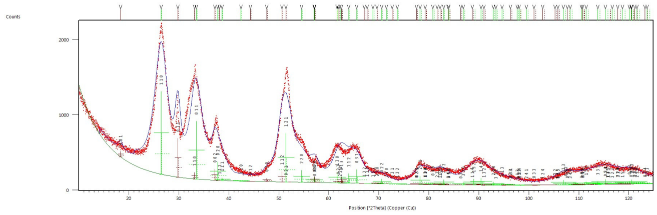

Figure S1. The Rietveld analysis of the xrd pattern obtained on the material not supported on the copper support, using SnO SG P4nmm (no. 129) and SnO_2 SG P42mnm (no. 136) as starting data for the model.

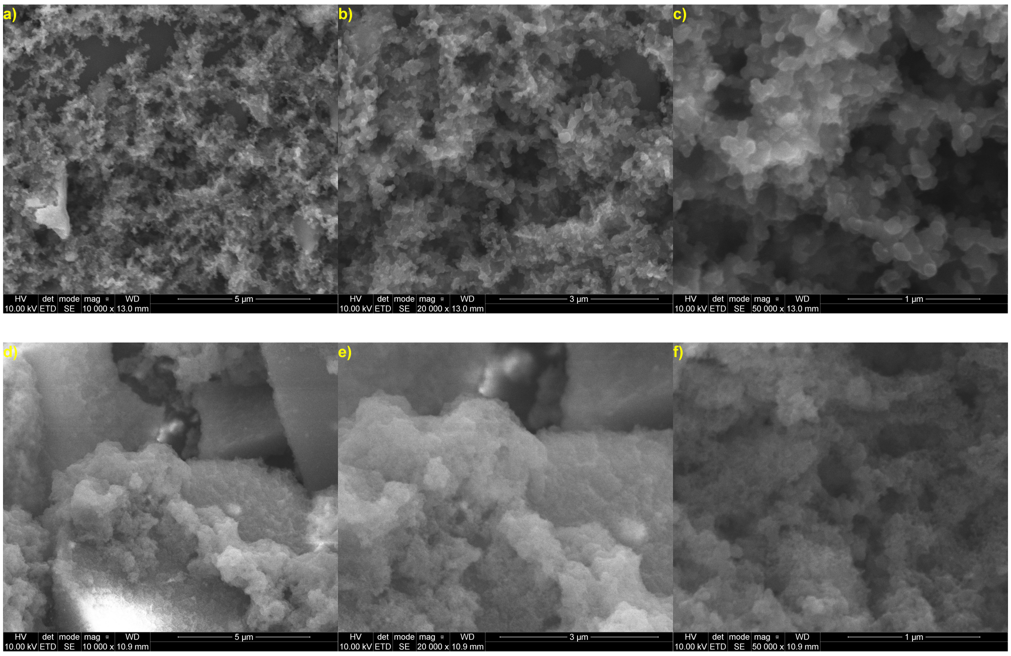

Figure S2. SEM image of SnO_x/CHI surface before (a-c) and after (d-f) electrochemical measurements.

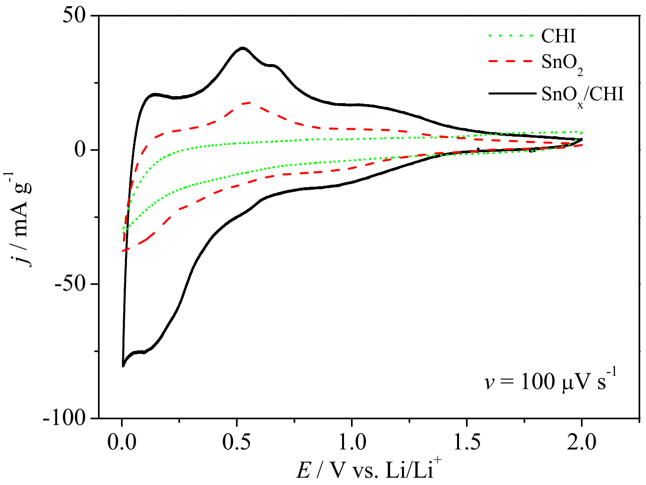

Figure S3. CV curves of CHI , SnO_2 and SnO_x/CHI electrode in LP30 at 100 V s^{-1}. The potential range: 0.005 V - 2.0 V vs. Li/Li^+.

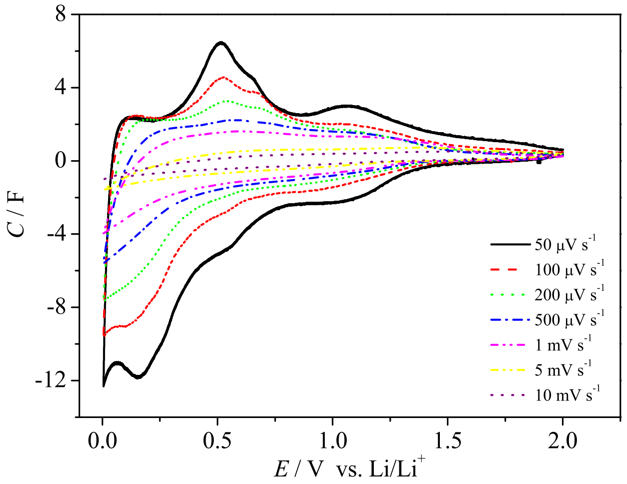

Figure S4. CV of SnO_x/CHI electrode material at a different sweep rates. Current is reexpressed as a capacitance.
